# Impact of cumulative fluid balance on the pharmacokinetics of extended infusion meropenem in critically ill patients with sepsis

**DOI:** 10.1186/s13054-021-03680-9

**Published:** 2021-07-17

**Authors:** Renata Černá Pařízková, Jiřina Martínková, Eduard Havel, Petr Šafránek, Milan Kaška, David Astapenko, Jan Bezouška, Jaroslav Chládek, Vladimír Černý

**Affiliations:** 1 Department of Anesthesiology, Resuscitation and Intensive Medicine, Charles University, Faculty of Medicine in Hradec Králové and University Hospital Hradec Králové, Sokolská 581, 50005 Hradec Králové, Czech Republic; 2 Department of Surgery, University Hospital Hradec Králové, Sokolská 581, 50005 Hradec Králové, Czech Republic; 3 Department of Surgery, Charles University, Faculty of Medicine in Hradec Králové and University Hospital Hradec Králové, Sokolská 581, 50005 Hradec Králové, Czech Republic; 4 Department of Pharmacology, Charles University, Faculty of Medicine in Hradec Králové, Šimkova 870, 50003 Hradec Králové, Czech Republic

**Keywords:** Meropenem, Pharmacokinetics, Pharmacodynamics, Critically ill patients, Sepsis, Fluid therapy

## Abstract

**Background:**

Meropenem dosing for septic critically patients is difficult due to pathophysiological changes associated with sepsis as well as supportive symptomatic therapies. A prospective single-center study assessed whether fluid retention alters meropenem pharmacokinetics and the achievement of the pharmacokinetic/pharmacodynamic (PK/PD) targets for efficacy.

**Methods:**

Twenty-five septic ICU patients (19 m, 6f) aged 32–86 years with the mean APACHE II score of 20.2 (range 11–33), suffering mainly from perioperative intra-abdominal or respiratory infections and septic shock (*n* = 18), were investigated over three days after the start of extended 3-h i.v. infusions of meropenem q8h. Urinary creatinine clearance (CL_cr_) and cumulative fluid balance (CFB) were measured daily. Plasma meropenem was measured, and Bayesian estimates of PK parameters were calculated.

**Results:**

Eleven patients (9 with peritonitis) were classified as fluid overload (FO) based on a positive day 1 CFB of more than 10% body weight. Compared to NoFO patients (*n* = 14, 11 with pneumonia), the FO patients had a lower meropenem clearance (CL_me_ 8.5 ± 3.2 vs 11.5 ± 3.5 L/h), higher volume of distribution (V_1_ 14.9 ± 3.5 vs 13.5 ± 4.1 L) and longer half-life (t_1/2_ 1.4 ± 0.63 vs 0.92 ± 0.54 h) (*p* < 0.05). Over three days, the CFB of the FO patients decreased (11.7 ± 3.3 vs 6.7 ± 4.3 L, *p* < 0.05) and the PK parameters reached the values comparable with NoFO patients (CL_me_ 12.4 ± 3.8 vs 11.5 ± 2.0 L/h, V_1_ 13.7 ± 2.0 vs 14.0 ± 5.1 L, t_1/2_ 0.81 ± 0.23 vs 0.87 ± 0.40 h). The CL_cr_ and Cockroft–Gault CL_cr_ were stable in time and comparable. The correlation with CL_me_ was weak to moderate (CL_cr_, day 3 CGCL_cr_) or absent (day 1 and 2 CGCL_cr_). Dosing with 2 g meropenem q8h ensured adequate concentrations to treat infections with sensitive pathogens (MIC 2 mg/L). The proportion of pre-dose concentrations exceeding the MIC 8 mg/L and the fraction time with a target-exceeding concentration were higher in the FO group (day 1–3 *f* C_min_ > MIC: 67 vs 27%, *p* < 0.001; day 1%*f* T > MIC: 79 ± 17 vs 58 ± 17, *p* < 0.05).

**Conclusions:**

These findings emphasize the importance of TDM and a cautious approach to augmented maintenance dosing of meropenem to patients with FO infected with less susceptible pathogens, if guided by population covariate relationships between CL_me_ and creatinine clearance.

**Supplementary Information:**

The online version contains supplementary material available at 10.1186/s13054-021-03680-9.

## Introduction

Meropenem, a potent ß-lactam antibiotic of the carbapenem group, is a drug commonly prescribed to critically ill patients with sepsis. The time-dependent bactericidal activity of meropenem requires that the pathogen be exposed to effective concentrations for a sufficient time. Therefore, the pharmacokinetic/pharmacodynamic (PK/PD) target for efficacy is the percent fraction time of the inter-dose interval with the concentration of free (i.e., protein-unbound) meropenem above the minimum inhibitory concentration of the given strain of bacteria (%*f*T > MIC) [1–3].

Renal excretion is a major elimination route for meropenem. Biomarkers of kidney function, including measured and calculated creatinine clearance (CL_cr_) or glomerular filtration rate (GFR) mathematical estimates, help to explain a part of the inter- and intraindividual variability in meropenem clearance (CL_me_) of critically patients in the intensive care unit (ICU) [4–6]. Acute kidney injury (AKI), which frequently develops in critically ill patients, creates the risk for accumulation of meropenem to high, potentially toxic, concentrations [7, 8]. By contrast, hyperdynamic circulation and augmented renal clearance of meropenem are present in septic patients in whom an adequate response to treatments with fluids and vasopressors has been achieved. This subpopulation of the ICU patients is at particular risk for sub-therapeutic plasma concentrations and non-attainment of the PK/PD target after standard dosing regimens with meropenem and other beta-lactams [9]. According to recent recommendations, the best way to overcome the remarkable variability of meropenem PK/PD in critically ill ICU patients is therapeutic drug monitoring (TDM) and pharmacokinetically guided dose adjustment [10, 11].

Excessive fluid therapy has been shown to cause worse outcomes of septic critically ill patients, including length of the ICU stay and mortality. Highly positive fluid balance further deteriorates diastolic dysfunction and capillary leak syndrome and leads to interstitial lung oedema, elevated renal venous pressure, reduced kidney perfusion and increased interstitial pressure [12]. Published studies with ICU patients did not examine in detail the impact of variable fluid balance on meropenem pharmacokinetics. The present prospective study aimed to assess whether fluid retention, brought about by early goal-directed fluid therapy of critically ill septic patients, may alter PK and achievement of PK/PD targets for efficacy in the early phase of antibacterial therapy with meropenem.

## Methods

### Study design

This prospective study was conducted at the Department of Surgery, of University Hospital and the Faculty of Medicine in Hradec Králové, Charles University in Prague, Czech Republic.

### Ethics statement

The study was performed in accordance with the Declaration of Helsinki (1964), including later amendments. The experimental protocol and informed consent were approved by the Ethics Committee of the University Hospital Hradec Králové, Czech Republic. The patients or their legal guardians signed informed consent before participating in the study.

### Patients and treatments

Critically ill patients admitted to the surgical ICU of the University Hospital in Hradec Králové were enrolled between 2013 and 2018. All patients suffered from a severe infection, were > 18 years of age and were not hypersensitive to meropenem. Subjects were excluded from the study if they showed evidence of chronic liver disease, chronic kidney disease, acute renal failure, were under renal replacement therapy, or if their stay in ICU was short (< 24 h).

Eligible patients received 1 g (*n* = 3) or 2 g (*n* = 22) meropenem in a 3-h i.v. infusion every 8 h. Early goal-directed fluid therapy with i.v. balanced crystalloids was initiated and continued if the patients remained fluid responsive. Daily fluid balance (DFB) was calculated as the difference between fluid intake and output. The cumulative fluid balance (CFB) was obtained as a sum of the DFBs [13]. The cutoff value of 10 for the percent ratio of day one DFB to the body weight at the admission time to the ICU was used as a threshold for categorization of patients into the groups with fluid overload (FO) or without (NoFO).

Noradrenaline was administered to those patients who remained hypotensive (MAP < 65 mm Hg) despite the initial fluid resuscitation. Low-dose furosemide was used to prevent long-term fluid overload. Its dose was titrated up in patients with signs of oliguria. The Acute Physiology and Chronic Health Evaluation II (APACHE II) and the Sequential Organ Failure Assessment (SOFA) scores were used to evaluate morbidity and to predict the outcome of septic patients [14, 15].

### Assessment of kidney function

Urinary clearance of creatinine (CL_cr_) was measured on every study day. Urine was collected over 24 h using an in-dwelling urinary catheter. In addition, the CL_cr_ was estimated using the Cockroft–Gault equation (CGCL_cr_) [16]. The extent of AKI was evaluated with the help of KDIGO **(**Kidney Disease Improving Global Outcomes**)** and RIFLE **(**Risk, Injury, Failure, Loss and End-stage Kidney Disease**)** criteria [17, 18].

### Sampling for pharmacokinetics and meropenem assay

The pharmacokinetic analysis was performed on days 1, 2 and 3 of the study, i.e., after the first, fourth and seventh dose. Blood collection was planned at the following intervals: 0.5 h pre-dose, and at 4, 4.5, 5.5, 6 and 7.5 h post-dose. Based on a local surge in the need for acute care in the ICU, the decision to reduce sampling frequency was left at the discretion of the physician.

Samples were placed immediately in an ice bath and centrifuged within 30 min at 3 000 rpm for 10 min. Two 0.5 mL plasma aliquots were mixed with 0.5 mL of 2-(N-morpholino)ethanesulfonic acid buffer as stabilizing solution. The mixture was immediately stored at − 20 °C before being transferred into − 80 °C (within two days) for long-term storage until analysis. Within two months from collection, the concentration of meropenem in plasma was determined using liquid chromatography-tandem mass spectrometry. Total meropenem concentration was assessed because the plasma protein binding of meropenem is negligible (2%) [19].

A modified version of the previously published method was adopted after an in-house validation [20]. Meropenem and deuterated internal standard (meropenem-d6) were extracted from plasma with the help of Waters Oasis HLB extraction cartridges and separated on a Discovery HS F5 column. The mobile phase was composed of 50% aqueous ammonium formate buffer (10 mM NH_3_, 0.1% formic acid) and 50% methanol (0.1% formic acid), and its flow rate was 0.3 mL/min. The analytes were detected using a triple quadrupole mass spectrometer with positive electrospray ionization. The quantification involved multiple reaction monitoring mode and the following of MRM transitions (*m/z*): 384 > 141 (meropenem-d6) and 390 > 14 (meropenem). The lower quantification limit was 0.05 mg/L. The range of linear response was from 0.87 to 209 mg/L. Accuracy and imprecision of meropenem determination in spiked quality control samples were 94.6–101.7% and 1.4 to 8.9%, respectively.

### Pharmacokinetic/pharmacodynamic analysis

The TDMx software was used for Bayesian estimation of pharmacokinetic parameters and prediction of the concentration–time profiles of meropenem in the plasma [21]. The population model of Li et al., implemented in TDMx predicts that the CL_me_ increases with the CGCL_cr_ and decreases with age (see Additional file 1) [22]. Estimates of PK parameters from intense sampling were compared to those from a limited sampling approach using two time points: the first sample taken within 4.5 h after the start of an infusion and the second one at either 7.5 h or 0.5 h pre-dose.

The concentrations at − 0.5 h before the start of the 4th and 7th infusions, and the post-infusion concentrations at 7.5 h after the first and 7th infusions were used for the calculation of the proportion of the assayed minimum concentrations of meropenem exceeding the target MICs (*f* C_min_ > MIC). The fraction time with a target-exceeding concentration of meropenem (%*f* T > MIC) was derived from the model predicted concentration–time profiles of plasma meropenem. Two PD targets were chosen: MIC = 2 mg/L and MIC = 8 mg/L, i.e., the MIC breakpoints for susceptible/intermediate (S/I) and intermediate/resistant (I/R) bacterial strains, such as *Enterobacteriaceae, Pseudomonas* spp. or *Acinetobacter* spp. [23].

### Statistical analysis

Statistical analysis was conducted with the help of GraphPad Prism 8 software (GraphPad Software, San Diego, CA, USA). Continuous data are presented as the mean (SD). Categorical data are presented as counts (%). Student´s *t* test, Mann–Whitney *U* test, one-way ANOVA for repeated measures or Fisher´s exact test for categorical data were used for comparisons between and within groups. Univariate and multivariate linear regression was used to examine the relationships between meropenem clearance and various fluid status or kidney function measures. Sample size for this study was established a priori as a convenience sample of 25 subjects. This sample size was estimated to be adequate for detection of a 40% difference in the mean CL_me_, supposing an inter-individual variability of 34% [22] and assuming 80% power and α of 5%.

## Results

### Patients and therapy

A total of 25 septic patients (19 males and 6 females) with perioperative intra-abdominal (*n* = 9) or respiratory (*n* = 12) infections and septic shock (*n* = 18) were enrolled in the study. Demographic and clinical characteristics are summarized in Table 1. According to initial scores of SOFA and APACHE II, most patients were classified to have moderate or severe disease. In the FO group of post-surgery patients, eight out of 11 patients suffered from intra-abdominal sepsis. The NoFO group was comprised of acutely ill medical and post-surgery patients with pneumonia-associated sepsis in 11 out of 14 patients. Treatment with noradrenaline and furosemide was comparable (Table 1). Microbiological findings are listed in the Additional file 1: Table S1.

**Table 1:** Characteristics of patients with sepsis

Characteristics	All patients	Fluid overload	No fluid overload
*N* (males/females)	25 (19/6)	11 (10/1)	14 (9/5)
Age (years)	67.0 (32–86)	68.2 (46–86)	66.0 (32–82)
Body weight (kg)	84.8 (59–120)	85.3 (70–113)	84.4 (59–120)
BMI (kg/m^2^)	28.7 (21.6–41.5)	28.0 (21.6–34.1)	29.2 (21.7–41.5)
Source of infection (*n*, %)			
Respiratory	12 (48%)	1 (9%)	11 (79%)
Intra-abdominal	9 (36%)	8 (73%)	1 (7%)
Soft tissue	4 (16%)	2 (18%)	2 (14%)
Septic shock (*n*, %)	18 (72%)	9 (82%)	9 (64%)
Noradrenaline (n, %)	21 (84%)	9 (82%)	12 (86%)
Median (IQR) dose	11.8 (2.6–29.5)	6.9 (0.4–36.9)	13.6 (2.8–29.5)
Furosemide (*n*, %)	24 (96%)	11 (100%)	12 (86%)
Median (IQR) dose	142 (81–220)	170 (125–253)	98 (67–208)
CL_cr_ (ml/min/1.73 m^2^)^a^	81.5 (21–174)	82.1 (21–174)	81.2 (26–157)
APACHE II	20.2 (11–33)	21.1 (12–27)	19.6 (11–33)
SOFA	7.4 (2–13)	7.0 (3–13)	7. 7 (2–12)
Surgery (*n*, %)	17 (68%)	11 (100%)	6 (43%)
Mechanical ventilation (*n*, %)	24 (96%)	11 (100%)	13 (93%)
Days of MV	9.3 (0–43)	4.4 (2–10)	12 (0–43)
Days of AT	9.2 (3–15)	11.5 (8–15)	7.2 (3–15)
Mortality	6 (24%)	1 (9%)	5 (36%)

Data are numbers, percentages or arithmetic means (ranges). Abbreviations: APACHE II the Acute Physiology and Chronic Health Evaluation II score, SOFA the Sequential Organ Failure Assessment score. a creatinine clearance measured on day 1, MV mechanical ventilation, AT antimicrobial therapy; a A severe decrease in the CL_cr_ (< 30 ml/min/1.73 m^2^) was found in two FO patients; glomerular hyperfiltration (CL_cr_ > 160 mL/min/1.73 m^2^ in men and > 150 in women) was detected in one patient from the No fluid overload group

### Fluid status and kidney function

Results of fluid status and kidney function monitoring over the three study days are summarized in Table 2. The CFB of the FO group markedly decreased with time whereas a trend towards its moderate increase was detected in the NoFO group. No significant between- or within-group differences were found in Scr, CL_cr_, CGCL_cr_ and 24-h urinary volume (Table 2). The total volume of urine collected over three days was higher in the FO group than the NoFO group (12.5 ± 2.8 vs. 8.9 ± 3.3 L, *p* < 0.01).

**Table 2:** Characteristics of fluid status and kidney function. Pharmacokinetic parameters of meropenem

Characteristics	Day	Fluid overload	No fluid overload
CFB (L)	1	11.7 (3.3)^2,3,#^	2.4 (1.8)^#^
	2	8.0 (4.3)^1,#^	3.3 (1.9)^#^
	3	6.7 (4.3)^1^	4.1 (2.6)
24-h urine output^a^ (L)	1	3.7 (2.1)	2.8 (1.1)
	2	4.0 (2.0)	3.1 (1.6)
	3	4.0 (2.1)	3.1 (1.2)
Scr^b^ (μmol/L)	1	91 (46)^2,3^	81 (36)
	2	71 (25)^1^	77 (27)
	3	69 (15)^1^	72 (28)
CL_cr_ (L/h)	1	4.9 (2.6)	4.9 (2.2)
	2	6.8 (2.6)	5.4 (2.7)
	3	6.1 (2.5)	5.6 (2.9)
CGCL_cr_ (L/h)	1	5.7 (2.1)	6.0 (2.1)
	2	6.9 (2.6)	6.3 (2.7)
	3	7.1 (2.5)	6.7 (2.9)
CL_me_ (L/h)	1	8.5 (3.2)^3,#^	11.5 (3.5)^#^
	2	10.9 (3.0)	12.2 (3.6)
	3	12.4 (3.8)^1^	11.5 (2.0)
Q_12_ (L/h)	1	18.4 (1.9)	18.2 (3.6)
	2	17.5 (2.6)	16.5 (3.0)
	3	17.6 (1.9)	19.2 (4.6)
V_1_ (L)	1	14.9 (3.5)^#^	13.5 (4.1)^#^
	2	14.3 (2.3)	14.1 (4.8)
	3	13.7 (2.0)	14.0 (5.1)
V_2_ (L)	1	14.0 (1.7)	13.1 (1.7)
	2	14.6 (1.9)	14.2 (1.8)
	3	13.8 (1.2)	13.2 (2.8)
t_1/2_ (h)	1	1.4 (0.63)^3,#^	0.92 (0.54)^#^
	2	0.96 (0.26)	0.86 (0.39)
	3	0.81 (0.23)^1^	0.87 (0.40)

Results of the Tukey–Kramer multiple-comparison test (*α* = 0.05): upper numbers indicate within-group differences between the monitoring Days 1, 2 and 3; # the between-group difference at the particular day. a The urine output rates were higher than the threshold defining oliguria (0.5 mL/kg/h) in all but two patients from the No fluid overload group who fulfilled the criteria for the stage 2 AKI according to KDIGO or the injury class AKI according to RIFLE; b All Scr values were less than 354 micromol/L, i.e., the threshold indicating stage 3 AKI (KDIGO) or the failure class AKI (RIFLE); Abbreviations: CFB cumulative fluid balance, CL_cr_ measured creatinine clearance, CGCLcr creatinine clearace estimated using Cockroft-Gault equation, CL_me_ meropenem total clearance, Q_12_ inter-compartmental clearance, V_1_ and V_2_ meropenem distribution volumes of the central and peripheral compartments, t_1/2_ biological half-life

### Meropenem pharmacokinetics

**Fig. 1:**
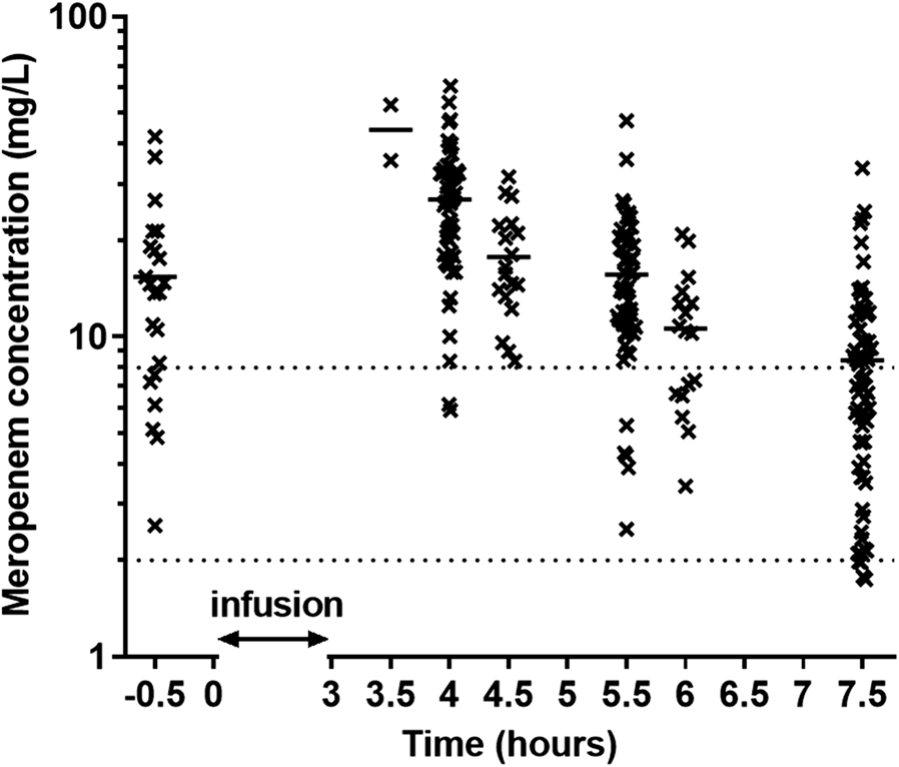
Assayed concentrations of meropenem vs. the sampling interval relative to the start of the last infusion. The patients were infused with 2 g meropenem as 3-h infusions every 8 h. For the 3 patients receiving  1 g, the concentrations recalculated to the dose of 2 g are shown. Solid lines are means and dotted lines depict concentrations equal to the MICs of 2 and 8 mg/L

A total of 235 blood samples were taken from 25 patients (median count per patient 9, range 6–12) following 75 infusions (median count per the inter-dose interval 3, range 2–5). The sampling intervals (relative to the start of the last infusion) and counts of blood specimens were as follows: pre-dose (− 0.5 h), *n* = 22; 4 h, *n* = 54; 4.5 h, *n* = 18; 5.5 h *n* = 53; 6 h, *n* = 17; 7.5 h, *n* = 67; other intervals, *n* = 4. The scatter plot of all assayed meropenem concentrations in the plasma samples is shown in Fig. 1.

Table 2 gives the statistical summary of individual pharmacokinetic parameters estimated using the Bayesian method. On the first day, the CL_me_ was significantly less and the V_1_ was larger in the FO group than in the NoFO group, resulting in a 1.5-fold longer half-life. From day 1 to 3, the CL_me_ of the FO group increased and the t_1/2_ dropped to values comparable with the other group. The average difference between the day one CL_me_ and CL_cr_ was twofold lower in the FO group than the NoFO group (2.8 vs. 5.6 L/h, *p* < 0.05), whereas day 3 results were comparable in both groups (5.0 vs. 5.1 L/h, Additional file 1: Table S2). The association between the CL_me_ and various fluid status or kidney function measures is summarized in Table 3. On day one, a moderate correlation was found by univariate linear regression between individual estimates of CL_me_ and either CFB, 24-h urine volume or CL_cr_. In a multivariate regression analysis, the independent predictors of the day one CL_me_ identified were CL_cr_ (*p* < 0.05) and CFB (*p* < 0.02). No correlation was found between the day one CL_me_ and CGCL_cr_. On day two, CL_me_ was found to be associated only with CL_cr_. On day 3, CGCL_cr_ was a stronger covariate of CL_me_, before CL_cr_ (Table 3).

**Table 3:** The association between the CLme and various fluid status or kidney function measures

Characteristic	Day	Correlation with meropenem clearance	
		r^2^	*p* value
CFB (L)	1	0.245	0.012
	2	0.001	0.95
	3	0.023	0.47
24-h urine output (L)	1	0.194	0.031
	2	0.006	0.70
	3	0.148	0.057
CL_cr_ (L/h)	1	0.306	0.004
	2	0.224	0.017
	3	0.238	0.014
CGCL_cr_ (L/h)	1	0.064	0.22
	2	0.063	0.23
	3	0.463	< 0.001

Abbreviations: see the legend to Table 2

In comparison with more intense sampling, Bayesian estimates of pharmacokinetic characteristics from two-timepoint concentrations showed the mean bias between − 5.1 and 1.4%, and imprecision values of less than 9.1% (Additional file 1: Table S3). The individually predicted concentrations of meropenem agreed well with the assayed concentrations, confirming the adequacy of both modeling methods (Additional file 1: Fig. S1, Table S4).

### Pharmacokinetic/pharmacodynamic target attainment

According to PK/PD indices *f* C_min_ > MIC and %*f* T > MIC, attainment of the lower target MIC of 2 mg/L was comparable in both groups, whereas for the MIC of 8 mg/L, the success rate was markedly higher in the FO group (Table 4).

**Table 4:** The PK/PD target attainment in the course of therapy with meropenem of patients with or without fluid overload on day 1

Characteristics	Day	Fluid overload	No fluid overload
*f* C_min_ > 2 mg/L (%)^a^	1–3	98 (89–100)	93 (80–98)
*f* C_min_ > 8 mg/L (%)^a^	1–3	67 (52–80)	27 (20–48)***
%*f* T > 2 mg/L (%)^b^	1	99 (2.3)	99 (2.7)
	2	100 (0.1)	98 (1.6)
	3	99 (1.2)	100 (0.1)
%*f* T > 8 mg/L (%)^b^	1	79 (17)^#,3^	58 (17)^#^
	2	78 (23)^#^	56 (13)^#^
	3	68 (21)^#,1^	58 (12)^#^

a the point estimate (95-% confidence interval) of the percent proportion, b the mean (standard deviation), *** *p* < 0.001 Fisher's exact test.; the results of the Tukey–Kramer multiple-comparison test (*α* = 0.05): upper numbers indicate within-group differences between the monitoring days 1, 2 and 3; # the between-group difference at the particular day.; abbreviations: fC_min_ > MIC the proportion of the assayed minimum concentrations of meropenem exceeding the targets of 2 mg/L and 8 mg/L; %f T > MIC the fraction time on the days 1, 2 and 3 with the concentration of meropenem exceeding the targets of 2 mg/L and 8 mg/L

## Discussion

To our best knowledge, this is the first study to investigate the CL_me_ and its covariate relationships in relation to the CFB of the ICU patients. The results support the idea that FO, defined as the CFB higher than 10% of the body weight at admission to the ICU, is associated with a decreased CL_me_. In addition, the t_1/2_ of the drug is longer in FO patients than NoFO patients who accumulate fluid less, due to the reduced CL_me_ and marginally enlarged meropenem distribution volume V_1_. In the FO group, gradual reduction of the CFB was accompanied by the increase in CL_me_, whereas the CL_cr_ and CGCL_cr_ were stable in time and comparable to the values of NoFO patients. Over the first three days of therapy with extended 3-h infusions of meropenem, dosing of 2 g every 8 h ensured adequate meropenem concentrations to treat infections with sensitive pathogens (MIC 2 mg/L). However, the success rate in the achievement of the PK/PD targets for pathogens with reduced antimicrobial susceptibility (MIC 8 mg/L) was substantially higher in the FO patients than NoFO patients. A CFB to admission weight-based definition of FO was used with the 10% cutoff, as this is associated with worse outcomes of critically ill patients including mortality [24, 25].

Numerous studies have described meropenem pharmacokinetics in critically ill ICU patients [4–7, 22, 26]. The estimates for the meropenem CL_me_ and V_d_ of our patients are within the range of values published for other cohorts of critically ill patients. However, the published population pharmacokinetic models differ and frequently produce biased and imprecise estimates of meropenem concentrations in external populations of critically ill patients. The causative factors are the differences in patient populations (demographic and clinical characteristics, origin and severity of infection, supportive treatments), meropenem dosing schedules, frequency and timing of blood collection relative to the start of dosing and the last dose, and last but not least, differences in meropenem assays and pharmacokinetic modeling methods. The moderate strength of the CL_me_ vs CL_cr_ relationships and the existence of many other factors influencing meropenem PK both emphasize the importance of meropenem TDM as a guide for individual dose adjustments in the highly vulnerable population of ICU patients. Development and validation of limited sampling strategies are an essential prerequisite for routine TDM of antibiotics in critically ill patients [11]. In the present study, Bayesian estimation using intense sampling as well as the two-timepoint concentrations approach resulted in an adequate agreement between individually predicted and assayed meropenem concentrations. Given the accuracy and precision of the estimates for PK parameters, exposure to extended infusion meropenem could be adequately assessed using the two point sampling.

Since meropenem is predominantly renally excreted, the main determinant of the exposure to the drug and of the PK/PD target attainment is renal function. The CGCL_cr_ is by far the most frequently studied covariate for CL_me_ in ICU patients [9, 10, 27, 28]. The population analysis of meropenem PK after standard dosing (1000 mg, 30-min infusion every 8 h) to critically ill surgical ICU patients examined 27 covariates and identified CGCL_cr_ as the only one with a pronounced impact on the probability of PK/PD target attainment [4]. In another study, the CGCL_cr_ was a better covariate than other serum creatinine-based equations, explaining approximately 50% of the inter-patient variability in the CL_me_ during continuous infusions of meropenem to ICU patients [28]. In contrast, the steady-state CL_me_ of surgical ICU patients continuously infused with meropenem was best predicted by the measured CL_cr_ from a 12-h urine collection, followed by the formulas containing serum cystatin C, and the least predictive covariate was CGCL_cr_ [26]. In agreement with results of others, we observed a weak correlation between the measured CL_cr_ and CL_me_. Of note, the CGCL_cr_ failed as a covariate on days 1 and 2 and showed an improved ability to predict CL_me_ on day 3, i.e., when fluid accumulation by the FO patients was markedly reduced.

An in-depth explanation of the reasons for the reduced CL_me_ of FO patients was hindered by the absence of simultaneous measurements of meropenem in urine and of the open-ring metabolite of meropenem in plasma and urine. Attenuation of tubular secretion of meropenem is one of the possible explanations, since the part of CL_me_ over CL_cr_ was markedly less in patients with FO on day 1, and the values of CL_cr_ were comparable in both groups. Organic anion transporters OAT1 and OAT3 involved in the tubular active transport of meropenem show a reduced expression and transport activity in response to deterioration of microcirculatory oxygenation and proximal tubular damage in early septic AKI [29, 30]. Alternatively, the glomerular filtration rate and the part of the CL_me_ governed by filtration could be reduced in FO patients. The GFR of the FO patients might have been overestimated by the CL_cr_ and CGCL_cr_, artificially lowering the estimated contribution of tubular secretion to the CL_me_. It has been previously shown that dilution by volume expansion can mask a true rise in serum creatinine, negatively affecting the accuracy of kidney function monitoring, and causing a delay in AKI detection and underestimation of its severity in critically ill ICU patients [31].

The lower CL_me_ of the FO patients implies a higher likelihood of achieving the PK/PD targets for meropenem after standard dosing. To avoid toxicity of the drug, incremental dosing with meropenem of critically ill patients infected with more resistant pathogens should be done with caution until the CFB is reduced. Another main message from the present study is that FO may also affect the relationships of the CL_me_ to the covariates CL_cr_ and CGCL_cr_, and adjustment of initial dosing guided by population covariate models.

Some limitations of the present study must be mentioned. Significant imbalances between patients with or without FO are potential source of bias. Patients with FO almost always had peritonitis and patients without predominantly had pneumonia. In patients with intraabdominal sepsis, intravascular fluid increasingly escapes to intraluminal and extraluminal spaces of the abdominal cavity, besides the interstitial space, and the achievement of adequate hemodynamic endpoints requires initial resuscitation with higher volumes of crystalloids [32]. A pulmonary source of infection together with a positive fluid balance can increase the risk for adult respiratory distress syndrome (ARDS), especially in septic shock [33]. Since fluid management was more restrictive and fluid accumulation on days 1 and 2 was less, most of the patients with a respiratory infection focus were allocated to the NoFO group. The PK data were collected from a low number of critically ill ICU patients in a single healthcare center. Therefore, the results and related hypotheses require confirmation in a larger study enabling population PK/PD modeling.

The strengths of this study include prospective design, measurement of urinary CL_cr_ and intensive PK sampling over three days, facilitating assessment of intraindividual changes in fluid balance in relation to the PK characteristics of meropenem.

## Conclusion

In conclusion, fluid retention in excess of 10% body weight in critically ill septic patients with FO is associated with a reduced clearance, prolonged t_1/2_ and a marginally increased distribution volume of meropenem. Over the first three days of therapy with extended 3-h infusions of meropenem (2 g, q8h), meropenem exposure and the success rate in the attainment of the PK/PD targets for pathogens with a reduced antimicrobial susceptibility (MIC 8 mg/L) were substantially higher in FO than NoFO patients with less positive CFB. Reduction of the CFB in the FO patients was accompanied by attenuation of the altered PK, whereas CL_cr_ and CGCL_cr_, the covariates of CL_me_, were stable in time and comparable between groups. These findings emphasize the importance of TDM and a cautious approach to augmented maintenance dosing of meropenem to patients with FO infected with less susceptible pathogens, if guided by population covariate relationships between CL_me_ and creatinine clearance.

## Supplementary Information

**Additional file 1.** The population model of meropenem used for calculation of Bayesian prior estimates in TDMx.

## Data Availability

The datasets used and/or analyzed during the current study are available from the corresponding author on reasonable request.
