# Supplementary material for: Impact of cumulative fluid balance on the pharmacokinetics of extended infusion meropenem in critically ill patients with sepsis
Source: Crit Care. 2021 Jul 17;25:251. doi: 10.1186/s13054-021-03680-9 (PMC8285835; doi:10.1186/s13054-021-03680-9)
Supplement: Supplementary file 1 — Additional file 1. The population model of meropenem used for calculation of Bayesian prior estimates in TDMx. [file 13054_2021_3680_MOESM1_ESM.docx]

**Supplementary materials**

The population model of meropenem used for calculation of Bayesian *prior* estimates in TDMx:

${CL}_{me} \left[ L/h \right]=14.6\times\left( \frac{{CGCL}_{cr}}{83} \right)^{0.62}\times\left( \frac{Age}{35} \right)^{-0.34}$

$$V_{1}\left[ L \right]= {10.8\times\left( \frac{TBW}{70} \right)}^{-0.99}$$

$$V_{2}\left[ L \right]= 12.6$$

$$Q_{12}\left[ L/h \right]= 18.6$$

Symbols: CL_me_…total clearance of meropenem, CGCL_cr_ …creatinine clearance estimated with Cockroft-Gault formula, V_1_, V_2_ …apparent meropenem distribution volumes of central and peripheral compartment, TBW…total body weight, Q_12_…intercompartmental clearance of meropenem

**Supplementary Table 1:** Bacterial isolates and sensitivity to meropenem

| Group | Bacterial isolates | Sensitivity |
| --- | --- | --- |
| FO | none | - |
| FO | none | - |
| FO | none | - |
| FO | *Klebsiela pneumoniae* | Yes |
| FO | *Klebsiela pneumoniae, Pseudomonas aeruginosa, Escherichia coli* | Yes |
| FO | *Klebsiela pneumoniae, Pseudomonas aeruginosa* | Yes |
| FO | *Escherichia coli, Enterobacter AMPC+* | Yes |
| FO | *Klebsiela pneumoniae, Acinetobacter* | Yes |
| FO | *Klebsiela pneumoniae ESBL+, Burghorderia multivoransY* | Yes |
| FO | *Pseudomonas aeruginosa* | Yes |
| FO | *Klebsiela pneumoniae ESBL+* | Yes |
| NoFO | none | - |
| NoFO | none | - |
| NoFO | none | - |
| NoFO | *Klebsiela pneumoniae* | Yes |
| NoFO | *Klebsiela pneumoniae, Pseudomonas aeruginosa, Escherichia coli* | Yes |
| NoFO | *Klebsiela pneumoniae, Pseudomonas aeruginosa* | Yes |
| NoFO | *Escherichia coli, Enterobacter AMPC+* | Yes |
| NoFO | *Klebsiela pneumoniae, Acinetobacter* | Yes |
| NoFO | *Klebsiela pneumoniae ESBL+, Burghorderia multivoransY* | Yes |
| NoFO | *Pseudomonas aeruginosa* | Yes |
| NoFO | *Klebsiela pneumoniae ESBL+* | Yes |
| NoFO | none | - |
| NoFO | *Enterobacter AMPC* | Yes |
| NoFO | *Klebsiela pneumoniae ESBL+, Escherichia coli* | Yes |

*Abbreviations: AMPC+…bacteria producing AmpC beta-lactamase, ESBL+…bacteria producing extended spectrum beta-lactamases*

**Supplementary Table 2** The part of meropenem total clearance (CL_me_) in excess of measured or calculated creatinine clearances. The differences between CL_me_ and measured creatinine clearance (CL_cr_) and, between CL_me_ and creatinine clearance estimated with Cockroft and Gault formula (CGCL_cr_).

| Characteristics | Day | Fluid overload | No fluid overload |
| --- | --- | --- | --- |
| CL_me_  - CL_cr_ (L/h) | 1 | 2.8 (3.2)^#,3^ | 5.6 (1.0) ^#^ |
|  | 2 | 3.6 (2.0) | 5.4 (1.6) |
|  | 3 | 5.0 (1.6)^1^ | 5.1 (1.0) |
| CL_me_  - CGCL_cr_ (L/h) | 1 | 2.9 (2.7) ^#,3^ | 4.3 (2.0) ^#^ |
|  | 2 | 3.4 (2.0) | 4.4 (2.3) |
|  | 3 | 4.4 (0.6) ^1^ | 3.9 (2.1) |

*Results of the Tukey-Kramer Multiple-Comparison Test (α=0.05): upper numbers indicate within-group differences between the monitoring Days 1, 2 and 3; #...the between-group difference on the particular day*

**Supplementary Table 3**. Bayesian estimates of meropenem pharmacokinetic parameters calculated with the help of two concentrations per dosing interval

| Characteristics | Day | Fluid overload | No fluid overload |
| --- | --- | --- | --- |
| CL_me_ (L/h) | 1 | 8.9 (3.4)^3, #^ | 11.7 (3.5) ^#^ |
|  | 2 | 10.5 (4.0) | 12.7 (3.3) |
|  | 3 | 11.6 (4.9)^1^ | 11.6 (2.0) |
| Q_12_ (L/h) | 1 | 18.3 (3.3) | 18.2 (3.3) |
|  | 2 | 18.6 (1.9) | 17.0 (3.0) |
|  | 3 | 18.1 (2.0) | 18.7 (3.3) |
| V_1_ (L) | 1 | 13.2 (1.2) | 13.4 (4.0) |
|  | 2 | 14.3 (2.4) | 14.0 (4.1) |
|  | 3 | 13.6 (2.3) | 13.5 (4.3) |
| V_2_ (L) | 1 | 13.5 (2.3) | 13.1 (1.7) |
|  | 2 | 13.6 (1.3) | 14.1 (1.8) |
|  | 3 | 13.3 (1.5) | 13.1 (2.0) |
| t_1/2_ (h) | 1 | 1.2 (0.43)^#^ | 0.91 (0.55)^#^ |
|  | 2 | 1.1 (0.56)^#^ | 0.86 (0.39)^#^ |
|  | 3 | 1.0 (0.54) | 0.87 (0.40) |

*Results of the Tukey-Kramer Multiple-Comparison Test (α=0.05): upper numbers indicate within-group differences between the monitoring Days 1, 2 and 3; #...the between-group difference at the particular day; Abbreviations: CL_me_…meropenem total clearance, Q_12_…inter-compartmental clearance, V_1_ and V_2_…meropenem distribution volumes of the central and peripheral compartments, t_1/2_…biological half-life*

**Supplementary Table 4.** Agreement between the observed and individually predicted meropenem concentrations with the help of Bayesian method involving all concentrations and two concentrations per dosing interval.

|  |  | Fluid overload | | | |  | No fluid overload | | | |
| --- | --- | --- | --- | --- | --- | --- | --- | --- | --- | --- |
|  |  | ME |  | RMSE |  |  | ME |  | RMSE |  |
| Day | Method | (mg/L) | (%) | (mg/L) | (%) |  | (mg/L) | (%) | (mg/L) | (%) |
| 1 | All concentrations | 0.48 | -1.6 | 2.7 | 12.9 |  | -0.01 | 0.33 | 1.9 | 12.7 |
|  | Two concentrations | 1.2 | 3.6 | 2.3 | 10.9 |  | 0.60 | 3.1 | 1.2 | 7.2 |
| 2 | All concentrations | 0.21 | 0.7 | 1.2 | 9.0 |  | 0.19 | 1.0 | 0.94 | 7.5 |
|  | Two concentrations | 1.2 | 7.2 | 2.5 | 11.4 |  | -0.20 | 0.31 | 1.2 | 9.5 |
| 3 | All concentrations | 1.0 | 3.1 | 1.7 | 9.9 |  | 0.08 | 0.8 | 1.2 | 9.1 |
|  | Two concentrations | 1.5 | 5.1 | 1.8 | 7.2 |  | 0.40 | 2.3 | 1.4 | 8.7 |

*Abbreviations: ME…mean error, RMSE…root mean square error*

**Supplementary fig. 1** Bayesian individual predictions calculated with the help of all concentrations (left figure) and two concentrations per dosing interval (right figure), plotted against observed concentrations of plasma meropenem. The dotted line is the identity line (Y=X).
